# Rectal Cancer Complicated by Acute Promyelocytic Leukemia

**DOI:** 10.7759/cureus.11118

**Published:** 2020-10-23

**Authors:** Shadi Alkhayyat

**Affiliations:** 1 Internal Medicine, King Abdulaziz University, Jeddah, SAU

**Keywords:** colorectal cancer, therapy related myeloid leukemia, acute promyelocytic leukemia, secondary, xelox

## Abstract

Globally colorectal cancer is one of the leading causes of mortality among men. Treatment modalities of chemotherapy and radiotherapy may put a risk in developing therapy related myeloid leukemia (TRML). TRML has been reported with Oxaliplatin, Capecitabine, and radiation therapy, which may consequently lead to DNA damage, causing a secondary disease. Acute Promyelocytic Leukemia (APL) is a type of TRML that occurs as a result of such therapies. We present a case of a 30 year old male who presented as a case of colorectal cancer. Subsequently, the patient received Capecitabine with radiotherapy followed by abdominoperineal resection. He received four additional cycles of XELOX protocol of chemotherapy. Months later, he returned with an intestinal obstruction and a picture of TRML. After a bone marrow biopsy, fluorescence in situ hybridization (FISH) and cytogenetics evaluation, the diagnosis of APL was confirmed. All-trans retinoic acid (ATRA) therapy was initiated with excellent response. In conclusion, TRML in the form of APL needs to be diagnosed early and treated as a medical emergency to prevent the high mortality rate associated with the disease.

## Introduction

Colorectal cancer (CRC), considered the third most leading cause of mortality globally, is most often driven from an array of heterogenous mutations and is considered a disease that is more prevalent in men than women, worldwide. Meanwhile, in the Middle East, it is the most common cancer diagnosed among the male population [1]. In Saudi Arabia, CRC are most often diagnosed at a late stage, which makes it one of the most common cancers among men with poor prognostic outcomes in the country [2].

Therapy related myeloid leukemia (TRML) is a clinical syndrome that has a latency of diagnosis between the exposure to cancer therapy and the development of leukemia, which ranges from several months to years. It occurs with agents that have DNA-damaging characteristics, such as cytotoxic chemotherapy and radiotherapy. Moreover, the most common agents that are associated with TRML are alkylating agents, radiation therapy, and topoisomerase II inhibitors [3]. TRML development due to these agents may be dose related, or due to individual susceptibility that might develop the unfortunate event of secondary TRML, with those having a combination of radiotherapy and chemotherapy having the highest risk of developing the syndrome. The most commonly reported primary malignancies that developed TRML are Non-Hodgkin lymphoma, lung and breast cancer. However, gastrointestinal incidence that had TRML developed after treatment are still rare to occur [4].

## Case presentation

Colorectal Cancer History

A 30-year-old male presented to the oncology clinic at a tertiary healthcare center in Jeddah complaining of painless rectal bleeding, which was progressive in nature. It became persistent with every bowel movement for a duration of ten weeks with no other associated symptoms. Upon further questioning, the patient revealed that he is a smoker for more than 10 years with no family history of malignancies. Regarding laboratory investigations, all initial parameters were within normal limits except for slight anemia and a mildly elevated carcinoembryonic antigen (CEA) of 7 ng/ml. Subsequently, the patient had a biopsy done in a different hospital setting, which showed invasive adenocarcinoma of the rectum. The patient then returned to the oncology clinic and a Computed Tomography Scan as well as a Magnetic Resonance Imaging of the pelvis was done, which showed rectal cancer with no evidence of distant metastasis (T3N1M0). Consequently, neoadjuvant therapy was started in the form of 25 cycles of radiotherapy along with chemotherapy in the form of Xeloda (Capecitabine) tablets 825 mg/m^2^, equal to 1500 mg in the morning and 1000mg in the evening.

The patient then underwent abdominoperineal resection surgery for tumor removal as no peritoneal metastasis evidence was found, with removal of 20 lymph nodes which were histopathologically negative for malignancy. Subsequently, he completed four cycles of six planned cycles of XELOX (Oxaliplatin and Capecitabine), which the patient refused to continue due to neuropathy, over a period of four months and was lost to follow up.

Therapy Related Myeloid Leukemia

**Figure 1:**
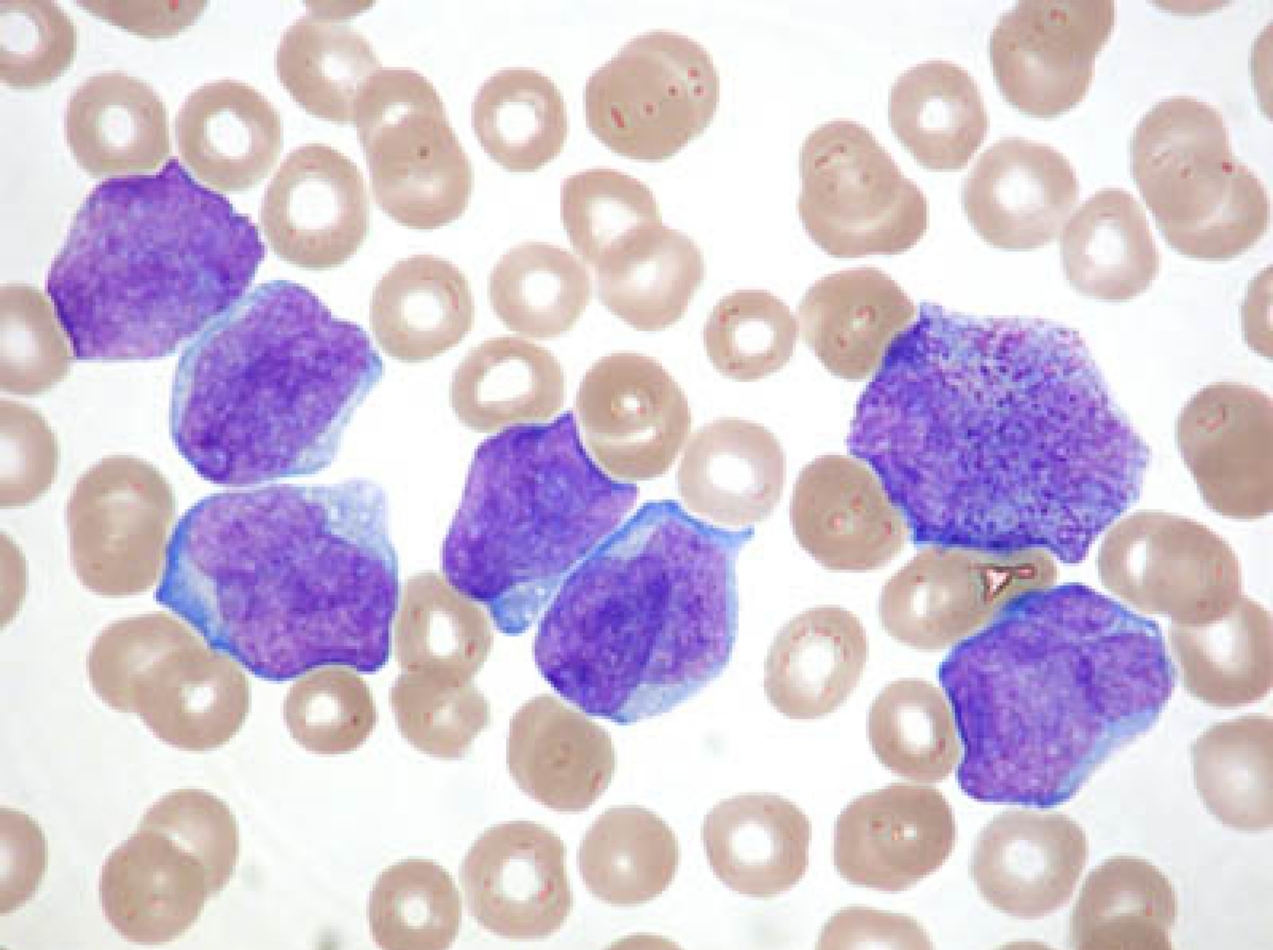
Multiple blast cells with reduced platelet

The patient presented to the emergency department six months after he was lost to follow, complaining of abdominal pain, distention, and bruising spots for few days. The abdominal pain was moderate in intensity and the ecchymosis was found in multiple areas throughout his body. Regarding his lab investigation, pancytopenia was seen with hemoglobin 8.9 g/dL, white blood cell counts 0.4 X 10^9^/L, and platelets 6 X 10^9^/L. Meanwhile, his International Normalized Ratio (INR) was 1.4 and his partial thromboplastin time was 16. Regarding his serology, hepatitis and immunodeficiency virus’s serology were negative. Furthermore, his liver and renal function tests were all within normal range. His abdominal ultrasound showed presence of peritoneal fluid and a picture of intestinal obstruction. After thorough evaluation by the surgical team, a mechanical obstruction was ruled out and he was admitted to the hospital accordingly for further evaluation. During his hospital stay, the patient had platelet transfusion and a repeated complete blood count showed rapid consumption with prolongation of INR (2) and a persistent thrombocytopenia. A hematological evaluation was done by blood smear exam under the microscope, which showed fragmented red blood cells supporting the diagnosis of a disseminated intravascular coagulation (DIC). In order to relieve the abdominal distension, an ascitic tap was performed and ascitic fluid analysis was positive for adenocarcinoma consistent with primary rectal cancer. Concurrently, as the patient was still having persistent pancytopenia, which was decreasing further with days, a bone marrow biopsy was performed which confirmed a diagnosis of Acute Promyelocytic Leukemia (APL) using blood film, Fluorescence In Situ Hybridization (FISH), and cytogenetics evaluation methods (Figure 1). The patient was treated as a TRML case of APL with all-trans retinoic acid (ATRA) by the hematology team. As a result, he had remarkable improvement after 48 hours with an improvement of his blood counts across all levels on the fifth day.

Lastly, as the patient was still positive for metastatic adenocarcinoma of CRC origin, we planned to get positron emission tomography testing, to be done after having the patient cleared and discharged by the hematology department. The intestinal obstruction improved with conservative treatment and after the initial ascitic aspiration. Unfortunately, as the patient was preparing for his discharge, he had a sudden episode of vomiting while eating his lunch, which was witnessed, and he became unresponsive, failed to recover even with aggressive resuscitation and passed away due to unknown reasons as no autopsy was done based on the request of the family.

## Discussion

APL is a form of acute myeloid leukemia and is considered one of the most fatal leukemias as it needs aggressive and timely treatment as a medical emergency [5]. They are often seen in middle-aged groups with a median age of 47 years and rarely does it occur in young adults [6]. According to Singh et al, the five years survival rate of APL is found to be less than 10% with the leading cause of death reported as intracranial hemorrhage as a result of the progressive rate of pancytopenia [7, 8]. Therefore, early management and treatment of this syndrome as a pure medical emergency can greatly differ in affecting the prognosis and survival of the patients. The incidence of a secondary APL to gastrointestinal malignancies is relatively rare, with only few reported cases. As such, colon involvement in the reported cases are found more than rectal involvement, which can be attributed hypothetically to the fact that colon malignancies are more common than rectal malignancies [9].

Furthermore, many chemotherapy treatments such as Oxaliplatin and Capecitabine have been shown to cause TRML, which are important in neoadjuvant therapy in cases of CRC and even gastrointestinal malignancies. Many reported cases have shown that Oxaliplatin may cause TRML with an initial presentation of pancytopenia, petechiae, or ecchymoses, which was similar to our patient [6, 10-12]. Such cases can present with rapid deterioration and complication such as DIC and associated hyperfibrinolysis, which can lead to severe hemorrhagic outcomes [6]. Moreover, mutations of chromosome 5 and 7 are frequently seen involved after Oxaliplatin therapy and a reason is yet unclear to the cause of such mutation [12]. However, Capecitabine works in a similar pattern, which is a DNA destructive pattern with intra- and inter-strand DNA cross links with leukemogenicity characteristics, where it disrupts replication and transcription of the DNA leading to apoptosis [11, 13].

Regarding the diagnosis of APL as a type of TRML, peripheral blood smear, and FISH are rapidly used as time is a sensitive matter in these cases. Furthermore, coagulopathy should be constantly monitored throughout the evaluation process with the option to use reverse transcriptase- polymerase chain reaction to aid in the diagnosis in APL [6]. Once suspicion is raised, ATRA is initiated as the mainstays in the treatment of such cases with supportive therapy and platelet transfusions to prevent DIC and hemorrhagic complications. If initiated early enough, up to 90% of patients can achieve complete remission and 75% can be disease free by a combination of ATRA and chemotherapy [14]. Studies have found that such treatment approach results in a cure rate of 80% - 90% of confirmed cases [15,16]. Therefore, educating the patients with such unfortunate events must be properly discussed when using such agents as it might prevent self-neglect and allow a proper follow up and monitoring to be done to diagnose such a complication early enough to prevent such unfortunate events.

## Conclusions

Therapy related myeloid leukemia in the form of Acute Promyelocytic Leukemia, secondary to chemotherapy such as Oxaliplatin and Capecitabine in colorectal cancer are relatively low. Proper patient education for follow-up and continuous monitoring is the key factor to diagnose and be able to intervene early enough to prevent the unfortunate events of late bleeding as it can cause severe coagulopathic and hemorrhagic complications.
